# Cardiovascular disease risk and all-cause mortality associated with accelerometer-measured physical activity and sedentary time ‒ a prospective population-based study in older adults

**DOI:** 10.1186/s12877-022-03414-8

**Published:** 2022-09-05

**Authors:** Miia Länsitie, Maarit Kangas, Jari Jokelainen, Mika Venojärvi, Markku Timonen, Sirkka Keinänen-Kiukaanniemi, Raija Korpelainen

**Affiliations:** 1 Department of Sports and Exercise Medicine, Oulu Deaconess Institute Foundation sr., Albertinkatu 18 A, 90100 Oulu, Finland; 2 Research Unit of Population Health, University of Oulu, Oulu, Finland; 3 Medical Research Center, Oulu University Hospital and University of Oulu, Oulu, Finland; 4 Northern Finland Birth Cohorts, Arctic Biobank, Infrastructure for Population Studies, Faculty of Medicine, University of Oulu, Oulu, Finland; 5 Unit of General Practice, Oulu University Hospital, Oulu, Finland; 6 Institute of Biomedicine, Sports and Exercise Medicine, University of Eastern Finland, Kuopio, Finland; 7 Healthcare and Social Services of Selänne, Pyhäjärvi, Finland

**Keywords:** Physical activity, Sedentary time, Accelerometry measurement, Cardiovascular disease, Mortality, Population-based

## Abstract

**Background:**

Low levels of physical activity (PA) and high sedentary time (ST) are common in older adults and lack of PA is a risk factor for cardiovascular disease (CVD). Knowledge about associations with accelerometer-measured PA, ST and CVD risk in older adults is insufficient. This study examines the associations of accelerometer-measured PA and ST with cardiovascular risk measured using the Framingham risk score (FRS) and all-cause mortality in older adults.

**Methods:**

A population-based sample of 660 (277 men, 383 women) older people (mean age 68.9) participated in the Oulu45 cohort study from 2013‒2015. PA and ST were measured with wrist-worn accelerometers at baseline for two weeks. Ten-year CVD risk (%) was estimated with FRS. The data for all-cause mortality were identified from the Digital and Population Data Services Agency, Finland after an average of 6.2 years follow-up. The associations between moderate to vigorous physical activity (MVPA), light physical activity (LPA), ST and FRS were analyzed using the multivariable linear regression analysis. Associations between LPA, ST and mortality were analyzed using the Cox proportional-hazard regression models.

**Results:**

Each 10 min increase in MVPA (β = -0.779, 95% CI -1.186 to -0.371, *p* < 0.001) and LPA (β = -0.293, 95% CI -0.448 to -0.138, *p* < 0.001) was negatively associated with FRS while a 10 min increase in ST (β = 0.290, 95% CI 0.158 to 0.421, *p* < 0.001) was positively associated with FRS. After adjustment for waist circumference, only ST was significantly associated with FRS. Each 10 min increase in LPA was associated with 6.5% lower all-cause mortality risk (HR = 0.935, 95% CI 0.884 to 0.990, *p* = 0.020) and each 10 min increase in ST with 5.6% increased mortality risk (HR = 1.056, 95% CI 1.007 to 1.108, *p* = 0.025).

**Conclusion:**

A higher amount of daily physical activity, at any intensity level, and avoidance of sedentary time are associated with reduced cardiovascular disease risk in older people. Higher time spent in light physical activity and lower sedentary time are associated with lower all-cause mortality.

## Background

Worldwide, almost 30% of the population does not meet physical activity (PA) recommendations [1]. Lack of physical activity is more common in older adults than in younger adults [1, 2]. Physical inactivity has been estimated to cause 6% of coronary heart diseases (CHD) and over five million deaths could be averted annually if all inactive persons become active [3].

More time spent in moderate-to-vigorous physical activity (MVPA) [4, 5] as well as in light [6] and total PA [4, 5] has been associated with lower incidence of cardiovascular disease (CVD) in middle-aged and older adults. In older adults, higher levels of MVPA [7] and light physical activity (LPA) [6, 7] as well as lower levels of sedentary time (ST) [7] have also been associated with lower incidence of CHD events. However, recommended levels of MVPA could attenuate increased CHD risk associated with ST [7].

The Framingham risk score (FRS) has been shown to predict coronary heart diseases (CHD) more accurately than the presence of metabolic syndrome [8]. In middle-aged adults, more time spent in MVPA has been associated with lower FRS regardless of length of time spent [9]. Accelerometer-measured ST is demonstrated to be positively associated with FRS for men [10]. A recent study suggests that adults with CVD or high CVD risk measured by FRS spend more time in ST and have lower MVPA levels than individuals with low CVD risk [11]. Another study using cluster analysis showed that inactive (highest ST, lowest LPA and MVPA) middle-aged women had higher CVD risk measured with FRS than women with at least moderate PA, while inactive men had higher CVD risk compared to very active men [12].

Higher levels of accelerometer-measured total PA, at any intensity level [7, 13–15] and lower ST [7, 14–16] have been associated with reduced risk of premature mortality in adults and older adults. However, in some studies, the association between LPA or ST and mortality has not been found after adjustment of MVPA [17]. High ST has been associated with higher mortality rates, especially for individuals with low MVPA [7, 18]. A recent study indicated that LPA could increase life expectancy, particularly for adults with low MVPA [19].

Accelerometers have been found to be reliable for measuring the amount and intensity of PA and ST [2, 20, 21]. Knowledge about dose–response associations with accelerometer-measured PA, especially LPA and ST, and health outcomes in older adults is insufficient [2, 21, 22]. However, associations between accelerometer-measured PA and incidents of CVD have been shown to be much stronger than reported in questionnaire-based studies [4, 17]. Wrist-worn accelerometers are well accepted and their use has been encouraged in population-based studies of older adults [2, 23]. There are no population-based studies on associations between accelerometer-measured PA, ST and FRS among older adults.

This study evaluates how accelerometer-measured PA and ST are associated with cardiovascular diseases risk, measured with the FRS, in a population-based sample of older adults. The study also aimed to reveal the associations between accelerometer-measured PA, ST and mortality in older adults.

## Methods

### Participants

This study is part of the Oulu45 cohort described in detail in our previous study [24]. Those participants born in 1945 in Oulu, Finland, who were still alive and whose addresses were known (*n* = 887) were invited to the follow-up study at the age of 67‒70 years (in 2013‒2015) and 714 (80%) participated. The final study population (*n* = 660, 92.4% of the total study population) of this prospective population-based study consisted of men (*n* = 277) and women (*n* = 383) whose PA and ST were successfully measured with accelerometer at baseline of the follow-up.

The baseline and follow-up study were approved by the Ethical Committee of the Northern Ostrobothnia Hospital District in Oulu, Finland (EETTMK 33/2001 and EETTMK 12/2013), and were undertaken in accordance with the Declaration of Helsinki. The participants provided their informed written consent for the study.

### Questionnaires

Cholesterol and hypertension medications were classified (no or yes) based on self-reported medications in use. Previously diagnosed coronary artery disease (CAD) and diabetes (type 1 or 2) were self-reported (no or yes). Smoking status was categorized as current smoker or non-smoker. An AUDIT questionnaire [25] was used to examine alcohol consumption and the total scores were calculated for those participants who answered all questions. Risky alcohol use was classified as ≥ 8 points.

Questions about perceived health (“How would you describe your health at the moment?”) and functional ability (“How would you describe your functional ability?”) were asked and answers dichotomized as satisfied (very good or pretty good) and non-satisfied (moderate, pretty poor, or very poor).

### Clinical examination and blood samples

The participants’ height (to nearest 0.5 cm) and weight (to nearest 0.1 kg) were measured. The waist circumference (WC) (to nearest 0.5 cm) was measured three times on the midpoint of the lowest rib and the iliac crest [26], and the mean of measurements was used in analyses. Blood pressure was measured twice (Omron M3, Omron Healthcare Europe BV, Netherlands) after 5 min’ rest in a seated position and the mean values of those measurements were used in the analyses. Visceral fat area was measured via bioelectrical impedance (InBody 720, InBody, Seoul, Korea).

Blood samples were taken after overnight fasting. The plasma cholesterol and triglycerides were analyzed immediately after the blood samples were taken. A standardized oral glucose tolerance test (OGTT) was performed after overnight fasting for participants without previously diagnosed diabetes or diabetes medication and whose plasma glucose levels were < 8.0 mmol/l measured using a blood glucose meter (Bauer Contour, Bayer Consumer Care AG). Diabetes was defined as plasma fasting glucose ≥ 7.0 mmol/l or 2-h glucose ≥ 11.1 mmol/l [27].

### Cardiovascular risk and register data

The FRS [28] was used to estimate the ten-year risk for CVD as a percentage. The FRS defines CVD risk including coronary death, myocardial infarction, coronary insufficiency, angina pectoris, heart failure, claudication, and ischemic and hemorrhagic stroke or transient ischemic attack. The FRS were calculated separately for all men and women from whom all the data needed were available. Age, HDL cholesterol, total cholesterol, systolic blood pressure (treated or not treated), smoking (yes/no), and diabetes mellitus (previously known diabetes or screen- detected type 2 diabetes on OGTT) (yes/no) were included in the FRS. Those participants whose FRS was > 20% were defined as high-risk individuals for CVD [28]. The FRS data was valid for 565 participants (21 missing values) without previously diagnosed CAD.

The date of deaths were requested from the Digital and Population Data Services Agency, Finland (until 14.10.2020) and participants were categorized as alive or dead. Follow-up time was calculated until death or until 14.10.2020 if the participant was alive.

### Physical activity

PA and ST were measured with a wrist-worn uniaxial accelerometer (Polar Active, Polar Electro Ltd., Kempele, Finland) at baseline in 2013–2015. Polar Active provides MET (metabolic equivalent) values (1 MET = 3.5 mL/kg/min) every 30 s to estimate energy expenditure of daily PA [29]. A strong correlation between energy expenditure measured by indirect calorimetry and Polar Active (*r* = 0.987) [30] has been demonstrated, and also with double-labeled water (*r* = 0.86) [31]. The participants were asked to wear the activity monitor for two weeks (24 h/day) on the wrist of their non-dominant hand. The activity monitor did not give feedback to the participants.

Daily averages (min/day) of accelerometer-measured PA were calculated for each participant and classified into five activity levels (very light 1.00–1.99 MET, light 2.00–3.49 MET, moderate 3.50–4.99 MET, vigorous 5.00–7.99 MET, and very vigorous ≥ 8.00 MET) using the limits set by the manufacturer. For the analysis, all activity with intensities between 1.00 and 1.99 MET was classified as ST, all activity with intensities between 2.00 and 3.49 MET as LPA, and all activity ≥ 3.50 MET was classified as MVPA. Previously, under free-living environment and in the most of the intensity levels, it has been shown that Polar Active is more comparable with hip-worn Actigraph GT3X monitor when using the standard Polar Active thresholds instead of using traditional thresholds (ST ≤ 1.5 MET, LPA 1.51–2.99 MET, MPA 3.00–5.99 MET and ≥ 6.00 MET) [32]. The cut-off values of PA intensity measured with Polar Active were developed for younger adults, which could affect the measurement reliability for older adults. All activity with intensities 1 MET or higher was calculated on wear time. Participants with at least four valid days (monitor wear time of ≥ 600 min/day) were included in the analyses. The first and last measurement days were excluded as incomplete. The mean of the valid measurement days was 13.

### Statistics

The results were analyzed using IBM SPSS Statistics software (SPSS 27 for Windows, SPSS Inc., Chicago, Illinois), and the statistical significance was set to *p* < 0.05. Differences among sexes were analyzed for categorical variables with the chi-square test and with independent samples t-test for continuous variables. Participants who reported diagnosed CAD were excluded when analyzing FRS.

The associations between MVPA, LPA, ST, and FRS were analyzed using the multivariable linear regression model. The models were built separately for MVPA, LPA and ST. Unstandardized regression coefficients (β) and 95% confidence intervals (CI) were estimated per 10 min and per 30 min increases in MVPA, LPA and ST. Based on the results of the univariate analysis, the models were adjusted for AUDIT score, living status (alone or with someone else) and monitor wear time (Model 1). Model 2 was adjusted for the same variables as Model 1 and also with WC. Furthermore, Model 1 for both ST and LPA were also adjusted with MVPA. Participants with previously diagnosed CAD were excluded in multivariable linear regression models for FRS. VIF < 5 was used in models for value of non-collinearity.

The Cox proportional hazards regression model was used to reveal the associations between LPA, ST, and all-cause mortality for the whole study population. In the Cox regression models, all participants who were alive when the follow-up ended (14.10.2020) and who had no missing values were considered as censored (*n* = 606). For the deaths, 28 events were included in the models. Hazard ratios (HR) and 95% CI were estimated per 10 min and per 30 min increases in LPA and ST. AUDIT score, blood pressure, blood pressure or cholesterol medication, smoking and living alone were not associated with mortality in univariate analysis. Based on univariate analysis FRS was included in the Cox proportional hazards regression model to describe the overall CVD risk. The models were also controlled for PA monitor wear time. MVPA was not included in the Cox proportional hazards regression models because it was not associated with mortality in univariate analysis.

## Results

The characteristics of study participants are presented in Table 1. Over 11% of study participants had self-reported previously diagnosed CAD and almost 17% had self-reported or screen-detected diabetes. Almost a half the participants had hypertension medication and almost every third used cholesterol medication. After the follow-up visit, 29 (4.4%) participants had died (see Table 1).

**Table 1:** Characteristics of the 660 study participants aged 67–70. Values are numbers (%)

	**Men** ***n* = 277 (42.0%)**	**Women** ***n* = 383 (58.0%)**	**All *n* = 660**
Deaths after the follow-up	20 (7.2)	9 (2.3)**	29 (4.4)
Hypertension medication	138 (50.0)	172 (45.0)	310 (47.1)
Cholesterol medication	91 (33.0)	110 (28.8)	201 (30.5)
Previously diagnosed CAD	43 (15.5)	31 (8.1)**	74 (11.2)
Previously known diabetes or screen-detected T2DM on OGTT	50 (18.4)	57 (15.3)	107 (16.6)
Current smoker	35 (12.9)	36 (9.5)	71 (10.9)
AUDIT score ≥ 8	59 (23.3)	16 (4.9)***	75 (12.9)
Self-reported health, good	146 (52.7)	249 (65.5)**	395 (60.1)
Self-reported functional ability, good	128 (46.4)	234 (61.1)***	362 (54.9)
Living alone	39 (14.7)	132 (35.7)***	171 (26.9)

*CAD* Coronary artery disease, *T2DM* Type 2 diabetes, *OGTT* Oral glucose tolerance test, *AUDIT* Alcohol use disorders identification test. Differences among sexes were analyzed using the chi-square test for categorical variables and the independent samples t-test for continuous variables. * *p* < 0.05, ** *p* < 0.01, *** *p* < 0.001. Values are calculated for the number of participants with data on the variable in question

Physical activity levels and risk factors for coronary artery disease are presented in Table 2. Men spent 42 min more sedentary time than women (*p* < 0.001) and amount of LPA was 13 min higher for woman (*p* = 0.034). There was no significant difference in MVPA among the sexes. CVD risk was high for 192 (83.8%) men and for 80 (23.8%) women (*p* < 0.001) (see Table 2).

**Table 2:** Physical activity and risk factors for coronary artery disease for the participants aged 67–70. Values are means (SD) unless otherwise stated

	**Men** ***n* = 277 (42.0%)**	**Women** ***n* = 383 (58.0%)**	**All** ***n* = 660**
ST min/day, mean (SD)	727 (113)	685 (99)***	703 (107)
LPA min/day, mean (SD)	246 (80)	259 (79)*	254 (80)
MVPA min/day, mean (SD)	43 (30)	39 (28)	41 (29)
Blood pressure, systolic	151 (19)	146 (20)**	148 (20)
Blood pressure, diastolic	87 (11)	86 (11)	87 (11)
BMI	27.9 (4.2)	27.5 (5.0)	27.6 (4.7)
WC	100 (12)	90 (13)***	94 (14)
VFA (cm^2^)	155 (46)	147 (26)*	150 (36)
Total cholesterol (mmol/l)	4.9 (1.1)	5.6 (1.3)***	5.3 (1.2)
LDL (mmol/l)	3.1 (1.0)	3.5 (1.1)***	3.3 (1.1)
HDL (mmol/l)	1.5 (0.4)	1.8 (0.4)***	1.6 (0.5)
Trigly (mmol/l)	1.3 (1.1)	1.2 (0.6)	1.3 (0.8)
Framingham risk scores	32 (14)	17 (10)***	23 (14)
High CVD risk > 20%, n (%)	192 (83.8)	80 (23.8)***	272 (48.1)

*BMI* Body mass index, *WC* Waist circumference, *VFA* Visceral fat area, *ST* Sedentary time 1.00–1.99 MET, *LPA* Light physical activity 2.00–3.49 MET, *MVPA* Moderate to vigorous physical activity ≥ 3.50 MET. Differences among sexes were analyzed using the independent samples t-test for continuous variables and the chi-square test for categorical variables. * *p* < 0.05, ** *p* < 0.01, *** *p* < 0.001. Values are calculated for the number of participants with data on the variable in question. For analyzing Framingham risk scores, participants with previously diagnosed coronary artery disease were excluded

**Fig. 1:**
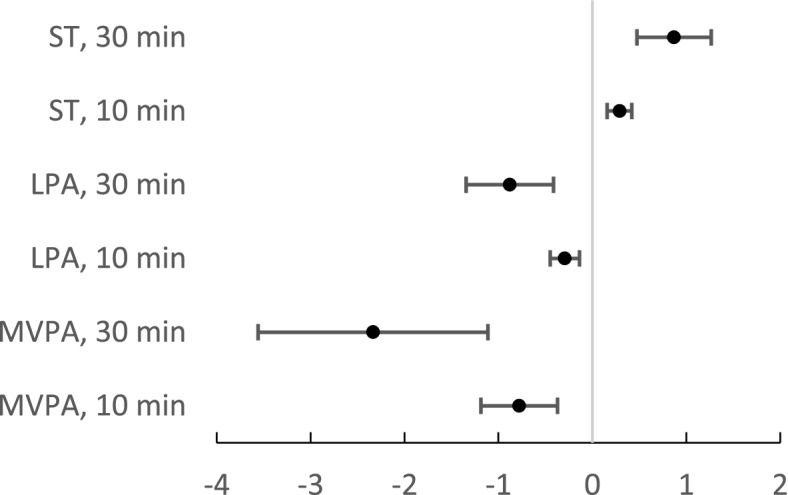
Framingham risk scores (FRS) for the participants without diagnosed coronary artery disease (*n* = 565). The lines represent the % change in FRS (the unstandardized regression coefficient, 95% CI) per each 10 or 30 min’ increase in sedentary time (ST), light physical activity (LPA), and moderate to vigorous physical activity (MVPA). Models were controlled for monitor wear time, living alone, and AUDIT scores

**Fig. 2:**
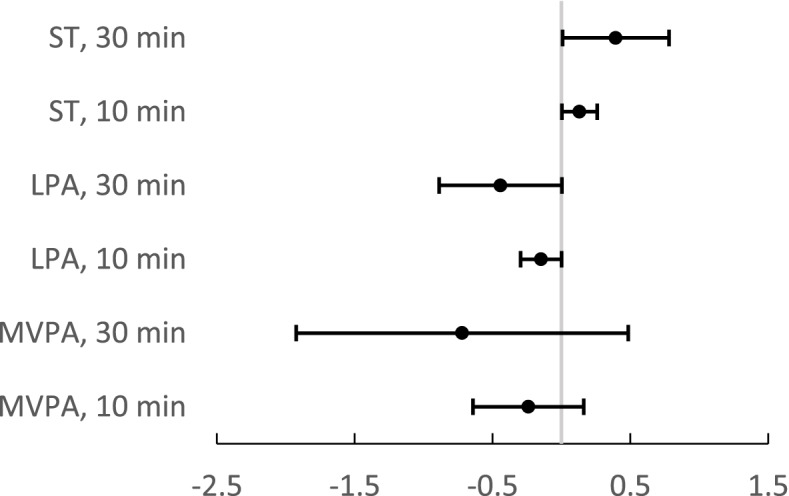
Framingham risk scores (FRS) for the participants without diagnosed coronary artery disease (*n* = 565). The lines represent the % change in FRS (the unstandardized regression coefficient, 95% CI) score per each 10 or 30 min’ increase in sedentary time (ST), light physical activity (LPA), and moderate to vigorous physical activity (MVPA). Models were controlled for waist circumference, monitor wear time, living alone, and AUDIT scores

The associations between MVPA, LPA, ST, and FRS according to the multivariable linear regression models are presented in Figs. 1 and 2. Every 10 min increase in MVPA (β = -0.779, 95% CI -1.186 to -0.371, *p* < 0.001) and LPA (β = -0.293, 95% CI -0.448 to -0.138, *p* < 0.001) was negatively associated with FRS and every 10 min increase in ST (β = 0.290, 95% CI 0.158 to 0.421, *p* < 0.001) was positively associated with FRS when monitor wear time, living status, and AUDIT scores were controlled for. Every 30 min increase in MVPA (β = -2.336, 95% CI -3.559 to -1.113, *p* < 0.001) or LPA (β = -0.879, 95% CI -1.345 to -0.413, *p* < 0.001) or 30 min increase in ST (β = 0.869, 95% CI 0.475 to 1.264, *p* < 0.001) having an equal but major association for FRS (see Fig. 1). When the Model 1 was adjusted also with sex, the association between LPA, MVPA, ST and Framingham risk score remained the same (data not shown) (Table 3).

**Table 3:** Factors associated with Framingham risk score among older people (*n* = 565) without diagnosed coronary artery disease

Variable	Regression coefficient (95% CI)	*p*-value*
**Model 1 for MVPA**		
MVPA, per 10 min increase	-0.779 (-1.186 to -0.371)	< 0.001
AUDIT score	0.501 (0.207 to 0.794)	0.001
Living alone	5.107 (2.437 to 7.777)	< 0.001
**Model 1 for LPA**		
LPA, per 10 min increase	-0.293 (-0.448 to -0.138)	< 0.001
AUDIT score	0.458 (0.163 to 0.754)	0.002
Living alone	5.345 (2.670 to 8.020)	< 0.001
**Model 1 for ST**		
ST, per 10 min increase	0.290 (0.158 to 0.421)	< 0.001
AUDIT score	0.444 (0.150 to 0.738)	0.003
Living alone	5.363 (2.702 to 8.024)	< 0.001
**Model 2 for MVPA**		
MVPA, per 10 min increase	-0.240 (-0.642 to 0.161)	0.241
AUDIT score	0.217 (-0.066 to 0.499)	0.132
Living alone	3.966 (1.455 to 6.478)	0.002
Waist circumference	0.387 (0.297 to 0.478)	< 0.001
**Model 2 for LPA**		
LPA, per 10 min increase	-0.148 (-0.296 to 0.001)	0.052
AUDIT score	0.190 (-0.093 to 0.472)	0.187
Living alone	4.103 (1.592 to 6.615)	0.001
Waist circumference	0.385 (0.297 to 0.473)	< 0.001
**Model 2 for ST**		
ST, per 10 min increase	0.131 (0.003 to 0.260)	0.046
AUDIT score	0.193 (-0.089 to 0.475)	0.180
Living alone	4.117 (1.606 to 6.629)	0.001
Waist circumference	0.378 (0.288 to 0.467)	< 0.001

*CI* Confidence intervals, *MVPA* Moderate to vigorous physical activity ≥ 3.50 MET, *LPA* Light physical activity 2.00–3.49 MET, *ST* Sedentary time 1.00–1.99 MET, *AUDIT* Alcohol use disorders identification test. Model 1 controlled for MVPA / LPA / ST, AUDIT score, living status and monitor wear time. Model 2 controlled for MVPA / LPA / ST, AUDIT score, living status, waist circumference and monitor wear time. *P-value for multivariable linear regression analysis. For MVPA, LPA and ST, the regression coefficient (95% CI) represents every 10 min increase in the examined variable

When the model was controlled also with WC (see Fig. 2), the positive association between every 10 min (β = 0.131, 95% CI 0.003 to 0.260, *p* = 0.046) and every 30 min increase in ST (β = 0.394, 95% CI 0.008 to 0.781, *p* = 0.046) and FRS remained the same. The association between LPA and FRS were of borderline significance (*p* = 0.052). There was no significant association between MVPA (*p* = 0.241) and FRS when WC was adjusted (see Fig. 2). When the Model 2 was adjusted also with sex, the association between ST, LPA and Framingham risk score remained the same. MVPA was negatively associated with Framingham risk score after adjustment sex (data not shown).

Furthermore, monitor wear time, living status, AUDIT scores, and MVPA were controlled for analyzing associations between ST, LPA and FRS. When the model was adjusted with ST and MVPA simultaneously (per 10 min increase for both), associations between ST and FRS remained similar (β = 0.216, 95% CI 0.054 to 0.378, *p* = 0.009) while there was no statistically significant association between MVPA and FRS. When the model was adjusted for both LPA and MVPA (per 10 min increases for both), associations between LPA and FRS (β = -0.233, 95% CI -0.385 to -0.061, *p* = 0.007) as well as MVPA and FRS (β = -0.598, 95% CI -1.024 to -0.172, *p* = 0.006) remained similar (data not shown on the figures).

**Fig. 3:**
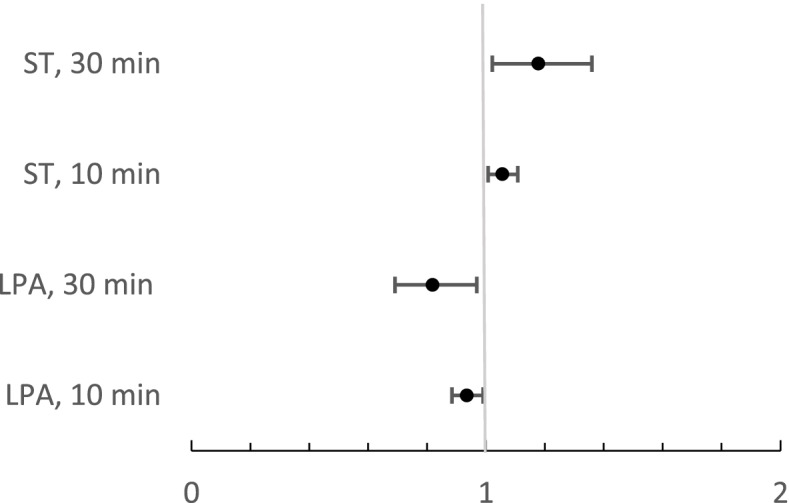
The hazard ratios (HR) (95% CI) for mortality for study participants (*n* = 660). The lines represent the change in HR per each 10 or 30 min’ increase in sedentary time (ST) and light physical activity (LPA). Models were controlled for Framingham risk score, and monitor wear time

The associations between LPA, ST, and all-cause mortality after 6.2 (min 0.6; max 7.7) years follow-up are presented in Fig. 3. Based on the results of the Cox regression model, every 10 min increase in LPA was associated with 6.5% lower mortality risk (HR = 0.935, 95% CI 0.884 to 0.990, *p* = 0.020) and every 10 min increase in ST with 5.6% increased mortality risk (HR = 1.056, 95% CI 1.007 to 1.108, *p* = 0.025). Every 30 min increase in LPA was associated with 18.1% lower mortality risk (HR = 0.819, 95% CI 0.691 to 0.969, *p* = 0.020), whereas every 30 min increase in ST was associated with 17.8% higher mortality risk (HR = 1.178, 95% CI 1.021 to 1.360, *p* = 0.025) (see Fig. 3). FRS was not significantly associated with mortality in Cox regression models. When the Cow regression models were adjusted also with sex, the association between LPA, ST and Framingham risk score remained the same (data not shown).

## Discussion

For the first time, this population-based study analyzed the association between accelerometer-measured physical activity and sedentary time and the risk of cardiovascular diseases measured by Framingham Risk Score in older adults. We found that more time spent in MVPA and LPA and less time spent sedentary were associated with lower CVD risk. Associations between LPA and ST with FRS were also found after adjustment of MVPA. However, after adjustment of WC only ST was associated with FRS. After an average of 6.2 years follow-up, more time spent in LPA and lower ST were also associated with all-cause mortality independently after adjusting CVD risk measured with FRS.

In this study, more time spent in MVPA or LPA and less ST were all associated with lower CVD risk measured with FRS. For MVPA and ST, the results are similar to previous findings with a younger population [9–11]. Previously, both LPA and MVPA were associated with lower CVD risk, measured with Reynold risk score in older women [33] which is also consistent with the findings of our study. When MVPA and LPA were both entered in the same model to describe the total PA level, the association between MVPA and FRS was over twice as strong as the association between LPA and FRS.

Based on our results, ST is an important and independent risk factor for CVD measured by FRS. When ST and MVPA were in the same model, only ST remained a statistically significant determinant of FRS there was no association between MVPA and FRS. Previously, high ST was shown to be associated with metabolic syndrome also in individuals with high cardiorespiratory fitness [34]. When WC was adjusted, only ST, not PA, was associated with lower FRS. Previously, low MVPA, but not ST were associated with high WC [35].

The results of this study are consistent with previous findings in that higher levels of accelerometer-measured LPA [7, 13–15] and lower levels of ST [7, 14–16] were associated with lower mortality rates. The associations between LPA, ST, and mortality were found regardless of CVD risk measured by FRS. However, in this study we found no association between MVPA and mortality, which is contradictory to previous studies with a larger sample size [7, 14, 15, 17]. In this study, there were only 29 deaths during the follow-up, which might explain the lack of association between MVPA and all-cause mortality. With such a small sample size, lack of association between MVPA and mortality may also be simply by chance. Associations between LPA, ST, and mortality could come out with small sample size because of higher levels of LPA and ST than MVPA.

Based on PA guidelines, older adults should spend 150 min per week in MVPA or should be at least as active as they can be if they have functional limitations [36, 37]. Adherence to PA recommendations even in later life has been shown to be associated with lower all-cause and cardiovascular mortality risk despite a previously inactive lifestyle [38]. However, the PA guidelines regarding MVPA for older adults could be too hard to achieve so our results about associations between accelerometer-measured LPA and ST to lower CVD risk and mortality is supportive. For public health, it might be the most important that older adults with high ST could at least increase LPA to promote their cardiovascular health and decrease the risk of mortality. A recently published review suggested that “sitting less and moving more” is practical and acceptable advice to decrease cardiovascular risk [39].

The strength of this study is its population-based, prospective nature. The target population was a homogenous, stable, and representative sample of aged men and women, obtained from the National Population Register of Finland, which has 100% coverage. Another strength of this study is accelerometer-measured PA and ST. The measurement period for PA in this study was approximately 13 days, instead of the usual one week. Thus, the PA measurement data could represent real-life conditions more accurately than shorter measurement periods. Wrist-worn accelerometers are well accepted in older adults and data is usually high quality [2].

There are some limitations in this study. In this study we used wrist-worn accelerometer. Previously, wrist-worn accelerometers were shown to be less precise to estimate ST in young adults compared to hip-worn monitors [40]. Another limitation of this study is that the most unwell participants may have dropped out of the study, which may introduce some bias to the results. On average, the participants attending the follow-up visit may be more active and healthier than the total 1945 birth cohort members, which may reduce the representativeness of the sample and the results of this study cannot be generalized to very ill or institutionalized older people.

A longer follow-up period might clarify the associations between MVPA, LPA, ST, and mortality, which were partly contradictory in this study compared to previous studies. In this study, we examined the associations between PA, ST, and all-cause mortality, not CVD mortality.

For a future studies, it is important to study associations between PA and ST, and CVD risk with larger study samples of older adults. It is also important to study how the length of PA and ST bouts are associated with risk of CVD and mortality in older people. After a longer follow-up, it might also be interesting to study associations with PA, ST, and CVD mortality.

## Conclusions

In conclusion, this prospective population-based study indicated that more time spent in PA and less time spent in ST were associated with lower CVD risk and also lower mortality rates in older people. Based on these results, a greater amount of daily physical activity, at any intensity level and avoiding sedentary time is recommended to reduce the risk of cardiovascular disease and all-cause mortality in old age.

## Data Availability

Oulu45 data is available from the University of Oulu, Infrastructure for Population Studies. Permission to use the data can be applied for research purposes via electronic material request portal. In the use of data, we follow the EU general data protection regulation (679/2016) and Finnish Data Protection Act. The use of personal data is based on cohort participant’s written informed consent at his/her latest follow-up study, which may cause limitations to its use. Please, contact NFBC project center (NFBCprojectcenter@oulu.fi) and visit the cohort website (www.oulu.fi/nfbc) for more information.

## Abbreviations

AUDIT: Alcohol use disorders identification test
BMI: Body mass index
CAD: Coronary artery disease
CHD: Coronary heart disease
CI: Confidence intervals
CVD: Cardiovascular disease
FRS: Framingham risk score
HR: Hazard ratios
LPA: Light physical activity
MET: Metabolic equivalent
MVPA: Moderate to vigorous physical activity
OGTT: Oral glucose tolerance test
PA: Physical activity
ST: Sedentary time
T2DM: Type 2 diabetes
VFA: Visceral fat area
WC: Waist circumference
